# Effect of Low-Molecular Organic Acids on the Migration Characteristics of Nickel in Reclaimed Soil from The Panyi Mine Area in China

**DOI:** 10.3390/toxics10120798

**Published:** 2022-12-19

**Authors:** Yonghong Zheng, Jiangwei Lu, Zhiguo Zhang, Yating Li, Yuning Tan, Weiqing Cai, Chengnan Ma, Fangling Chen

**Affiliations:** 1 School of Earth and Environment, Anhui University of Science and Technology, Huainan 232001, China; 2 Anhui Engineering Laboratory for Comprehensive Utilization of Water and Soil Resources and Ecological Protection in Mining Area with High Groundwater Level, Huainan 232001, China; 3 National Engineering Laboratory for Protection of Colliery Eco-Environment, Huainan 232001, China; 4 Institute of Energy, Hefei Comprehensive National Science Center, Hefei 230031, China

**Keywords:** heavy metal, nickel, citric acid, malic acid, Tessier chemical extraction

## Abstract

This study investigated the effects of low molecular weight organic acids (citric acid and malic acid) on the migration properties of nickel in soil. A reclaimed soil sample was obtained from the Panyi Mine in Huainan City, China. The effects of adding different concentrations of Ni, citric acid (CA) and malic acid (MA) were assessed on the migration and transformation of soil Ni forms. The results showed: (1) An increase in soil Ni activity with increasing Ni concentrations. (2) An increased proportion of exchangeable forms of Ni in soil with increased malic acid and citric acid concentrations, effectively promoting Ni mobility. In addition, the active Ni fraction in reclaimed soil increased significantly with increasing concentrations of citric and malic acid. The nickel activation effect of citric acid was found to be higher than that of malic acid. (3) The activation effect of organic acids on Ni weakened with aging, exhibiting a gradual transformation from the loosely bound form of Ni, to the strongly bound form. The results of this study provide a theoretical basis for improving the effectiveness and efficiency of the phytoremediation techniques used for the treatment of Ni-polluted soils.

## 1. Introduction

With economic development, mining resources have become indispensable to societies in many regions worldwide. However, mining activities have resulted in environmental pollution, particularly heavy metal pollution, which not only contaminates soil and groundwater resources but also affects air quality [1,2]. In addition, heavy metal elements can enter the food chain and present a direct or indirect risk to human health [3]. Excessive intake of nickel can cause toxicity. It has been reported that workers in industrial nickel refining facilities are more prone to lung cancer and nasopharyngeal cancer, with most patients presenting with skin inflammation and respiratory dysfunction as the first symptoms, conditions that seriously affect the quality of life of the patients [4]. Coal mine resources are generally rich in nickel (Ni), which may increase the risk of Ni release from mineral resources to the surrounding environment during mining activities, resulting in Ni soil pollution in agricultural land surrounding mining areas. In reclaimed soil areas filled with coal gangue, long-term soil cover has been found to result in higher soil Ni concentrations as compared to the background Ni content of soil in Huainan City and greater China [5], posing a threat to human health. According to reports, soil Ni concentrations exceed the background content in about 4.8% of the total surface of China, with levels second only to cadmium (Cd). In fact, soil Ni pollution has been shown to be insidious, cumulative, and irreversible. Chinese soil contamination risk control standards for agricultural land specify that as the pH of Huainan soil is between 6.5 and 7.5, the maximum allowable total nickel concentration must not exceed 100 mg/Kg [6].

In recent years, many remediation technologies have been used to address the issue of soil pollution, including physical remediation, chemical remediation, microbial remediation, and phytoremediation [7,8,9]. Although engineering measures are often used in physical remediation processes, they are costly, require copious materials, and are likely to cause significant damage to the soil structure [10]. In contrast, the organic complexation method used in chemical remediation approaches is cost-effective and time-efficient, but can generate organic pollution, preventing the widespread utilization of this technology [11,12]. Microbial remediation is capable of fixing relatively low heavy metal concentrations and has poor metabolic capacity [13]. However, phytoremediation has been found to be a low environmental impact remediation technique [14], providing a sustainable approach to bioremediation. This technique can be applied in situ in combination with other remediation technologies to significantly improve process efficiency for the removal of heavy metals [15]. However, heavy metal components can react with some soil substances, leading to changes in the physicochemical properties of the soil, affecting plant growth and the stability of soil ecosystems [16]. Therefore, it is important to comprehensively understand the reaction products formed during the remediation of soils contaminated with heavy metals [17]. Organic acids are utilized as natural chelating compounds [18,19,20], forming complexes with heavy metal elements and improving the efficiency of heavy metal removal from soil [21,22].

In the present study, Ni contamination of soils was investigated using reclaimed soil samples collected from mining areas in Huainan, China. In addition, the effects of exogenous heavy metals and endogenous organic acids on the morphology and migration of Ni in reclaimed soils were investigated. The methods and results of this study provide insight and will help guide the improvement of soil reclamation and management in Ni contaminated mining areas in Huainan City, China.

## 2. Materials and Methods

### 2.1. Soil Sampling and Preparation

Soil samples were collected from the top layer of soil on reclaimed land in the Panyi mine in Huainan, China. Soil samples were first spread evenly to remove biomass, debris, gravel and other impurities, then air-dried naturally without exposure to direct sunlight. The air-dried soil samples were passed through an 18-mesh sieve (particle size < 1 mm), then stored for subsequent use. The N, P, K, and organic matter content of the soil were measured according to the methods of Bao et al. (2000) [23]_._

### 2.2. Experimental Procedure

#### 2.2.1. Preparation of Ni-Contaminated Soil

According to the control standards of Soil Environmental Quality of Agricultural Land and Soil Pollution Risk (GB15618-2018) and Soil Environmental Quality Construction Land and Soil Pollution Risk (GB36600-2018), solutions containing varying Ni concentrations were added to reclaimed soil samples, forming soil concentrations of 100, 200, 300, and 600 mg/kg. The soil moisture content in Huainan is typically 30–50% [24], so the water content of the soil was kept within this range by the addition of tap water every 24 h. Soil samples were placed in glass petri dishes and transferred to an artificial climate incubator (RCX-180F). During the 7-day period, the artificial climate incubator maintained constant conditions, with a temperature of 25 °C and continual light exposure (the 24-h light was set by placing the sample in the incubator for 24 h of light), followed by air-drying to form the final Ni-contaminated soil samples.

#### 2.2.2. Organic Acid Addition

In this study, the effects of different types and concentrations of low molecular-weight organic acids on Ni morphology in soil environments were assessed by adding different concentrations of citric and malic acids to the prepared soil sample (Table 1). The organic acid-spiked soil samples were incubated for 7 days at 25 °C, then air-dried and sieved to collect particle sizes < 1 mm. All samples were prepared in triplicate. The effect of low molecular weight organic acids on the Ni formation in soil was evaluated under different incubation periods. A 12 mL volume of 10 mmol/L citric acid or malic acid was added to 20 g of soil samples contaminated with 300 mg/kg Ni, and the spiked soil samples were incubated for 1, 3, 5, 7, 15, and 30 days at 25 °C, then finally air-dried and sieved.

### 2.3. Analytical Methods

The soil physicochemical properties were determined according to standard soil analysis methods [25]. The total heavy metal concentration was measured at 120 °C by digesting 0.2 g of the sample with a mixture of HNO_3_/HClO_4_/HF acid (10 mL nitric acid, 2 mL hydrofluoric acid, and 3 mL perchloric acid). A modified five-step Tessier method was used to extract different forms of the Ni-contaminated soil, namely exchangeable (F1), carbonate-bound (F2), iron and manganese oxide-bound (F3), organic matter-bound (F4), and residual (F5) fractions [26,27]. Available fractions of Ni in the soil include the exchangeable and carbonate-bound forms, while non-available Ni forms include the Fe-Mn oxide-bound, organic-bound, and residual forms [28]. The Ni fractions in the test solution were analyzed by ICP-MS (PE NexION 300X; Perkin Elmer, Waltham, MA, USA).

### 2.4. Quality Control and Processing of Data

The chemicals and reagents used in all experiments were of analytical quality. Ni standard solutions were prepared using nickel nitrate (Ni (NO_3_)_2_), with calibration of different Ni fraction forms in contaminated soil exhibiting a relative deviation range of 0.55–3.05% (Table 2).

Experimental results were analyzed and graphs were plotted using SPSS v.26 and Origin 2021 software, respectively.

## 3. Results

### 3.1. Physicochemical Properties of Coal Mine Reclamation Soil

The physicochemical properties of the reclaimed soil sample are summarized in Table 3. Compared with the second national soil census nutrient-grading standard [29], the average pH value of the soil in the reclaimed area of the Panyi mine was alkaline, with a pH value of 7.80, indicating that the soil would have a slightly inhibitory effect on plants [30]. The mean value of soil bulk density was 1.33 g/cm^3^, indicating that the soil is compacted due to human activity in this area. In addition, the average soil organic matter content was 4.13 g/kg. According to the second national soil census nutrient-grading standard, the soil organic matter content was extremely deficient. In contrast, the average soil contents of available potassium, phosphorus, and nitrogen were 191.03 mg/kg, 10.41 mg/kg, and 28.98 mg/kg, respectively. These findings suggest that there were high available potassium and phosphorus concentrations in the reclaimed soil, while the residual content of these components was low.

### 3.2. Distribution of Ni Forms in Soil Contaminated with Exogenous Ni

**Figure 1:**
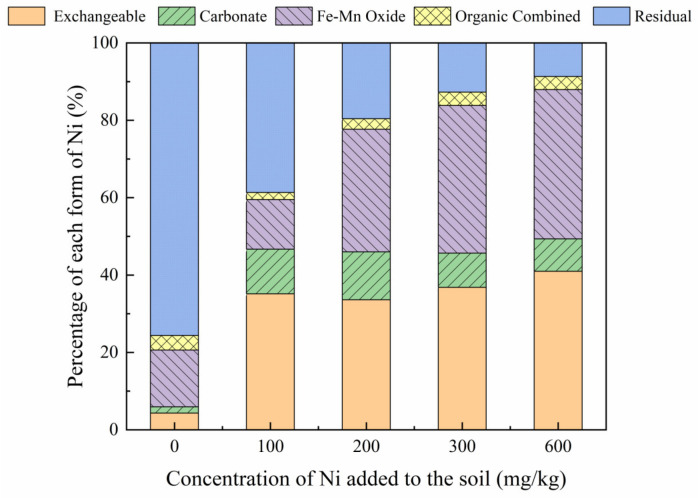
Distribution of different Ni forms after the addition of exogenous Ni (0, 100, 200, 300, 600 mg/kg) to the mine soil.

The distribution patterns of Ni in soil containing increasing Ni concentrations are shown in Figure 1. Before adding Ni to the reclaimed soil, the main soil Ni forms were residual Ni, Ni combined with iron-manganese oxide, exchangeable Ni, Ni carbonate, and Ni combined with organic matter, accounting for 76.53%, 14.69%, 4.33%, 1.60%, and 3.75% of the total Ni concentration, respectively. However, the addition of Ni to the reclaimed soil resulted in a change in the proportions of soil Ni fractions, with a significant increase in the Ni exchangeable form from 4.33% to 40.99%, making it the major Ni form in the soil. This result may be due to the short incubation time and the water-soluble ion-exchange form used. Moreover, the exchangeable Ni form is weakly adsorbed to the soil and has strong mobility, facilitating its release. The addition of Ni resulted in a significant decrease in the residual Ni from 75.63% to 8.69%, while increasing the abundance of iron-manganese oxide and carbonate forms from 14.69% to 38.58% and 1.60% to 8.36%, respectively. Therefore, the addition of Ni to the soil promoted the combination of Ni with soil carbonate minerals and increased the soil Ni concentrations, increasing the content associated with iron and manganese oxides. Compared with these forms, Ni ions can undergo complexation reactions with organic matter in the soil, slightly increasing the proportion of organically bound Ni. Residual Ni mainly exists in soil lattices, including silicates and primary or secondary minerals, resulting in the stable chemical properties of residual Ni, which is not easily affected by changes in the soil environment. The proportion of residual Ni decreased from 75.63% to 8.69% with increasing Ni concentrations. These results show that the addition of different concentrations of Ni to the soil can significantly change the distribution of Ni fractions in the soil, indicating that the forms of Ni in the soil were equally influenced by both the total amount of Ni and the added concentration of exogenous Ni.

### 3.3. Influence of Organic Acids on The Form of Ni in Soils

#### 3.3.1. Effect of Citric Acid on Ni Form

**Figure 2:**
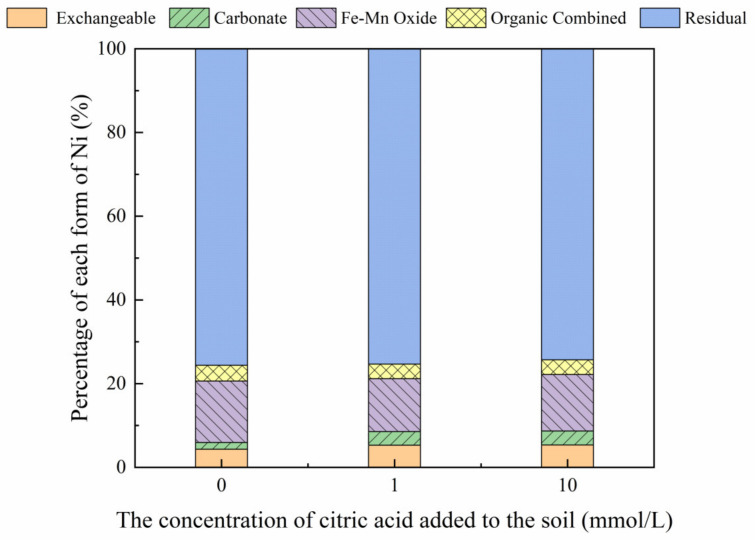
Distribution of different Ni forms after the addition of citric acid (0, 1, 10 mmol/L) to the air-dried mine soil.

Results showed that the distribution of soil Ni contents changed following the addition of different concentrations of citric acid (Figure 2). According to the results, the addition of increasing citric acid concentrations increased the proportion of exchangeable Ni and carbonate forms from 4.33% to 5.33% and 1.60% to 3.34%, respectively. In addition, increasing citric acid concentrations decreased the proportion of the iron-manganese oxide, organic, and residual forms from 14.69% to 13.49%, 3.75% to 3.50%, and 75.63% to 74.33%, respectively. These results demonstrate that citric acid exerted a minor effect on soil Ni forms.

**Figure 3:**
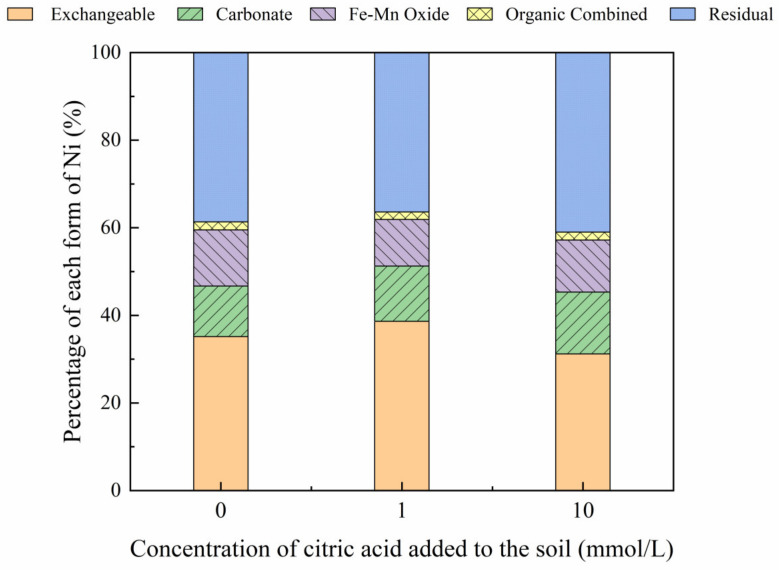
Distribution of different Ni forms in Ni-100 soil after the addition of citric acid (0, 1, 10 mmol/L).

**Figure 4:**
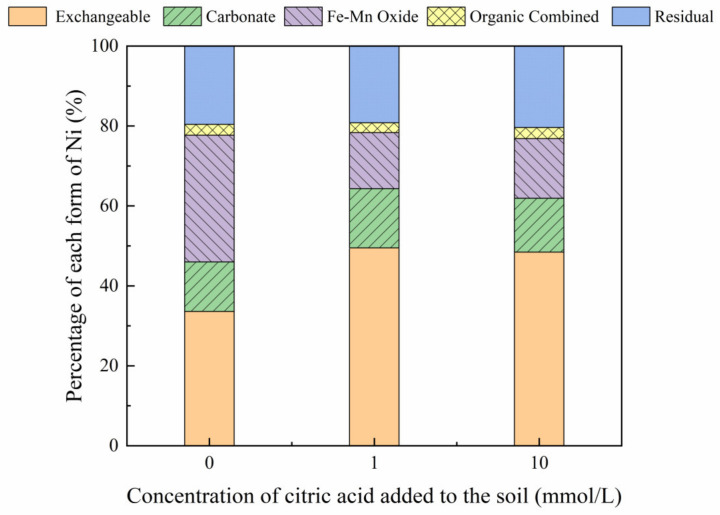
Distribution of different Ni forms in Ni-200 soil after the addition of citric acid (0, 1, 10 mmol/L).

**Figure 5:**
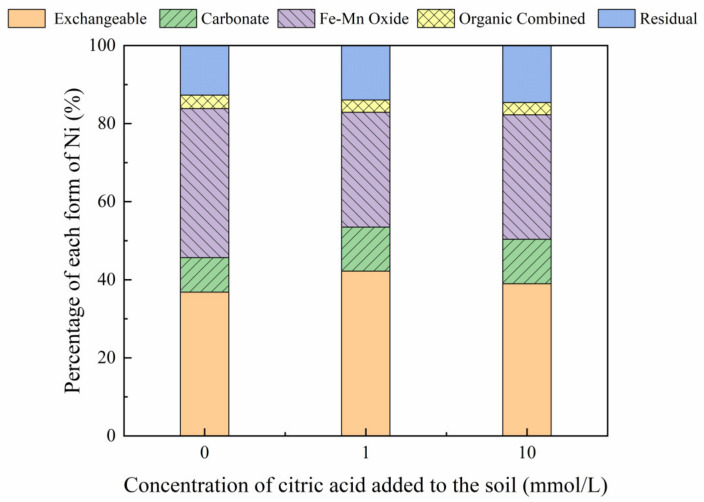
Distribution of different Ni forms in Ni-300 soil after the addition of citric acid (0, 1, 10 mmol/L).

**Figure 6:**
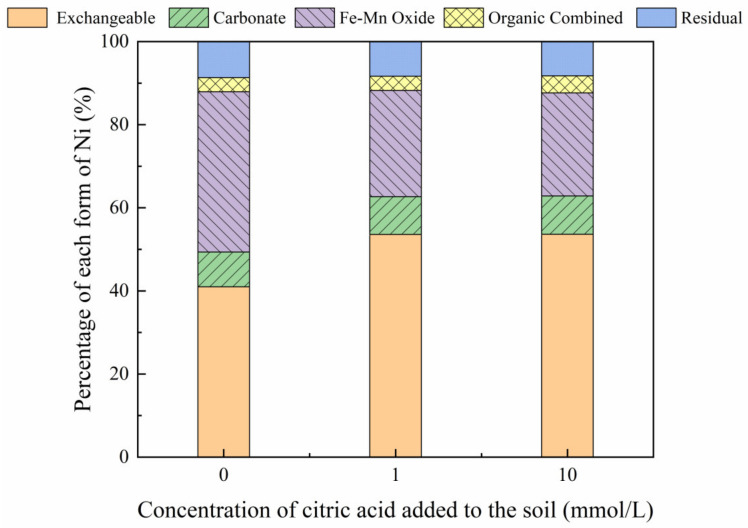
Distribution of different Ni forms in Ni-600 soil after the addition of citric acid (0, 1, 10 mmol/L).

Figure 3, Figure 4, Figure 5 and Figure 6 show the changes in Ni forms in the reclaimed soil following the addition of increasing concentrations of citric acid. The results revealed significant variations in the exchangeable Ni form as a result of increased citric acid concentrations, with this effect especially apparent at a soil concentration of 200 mg/kg, with an increase in the proportion of exchangeable Ni from 33.60% to 48.46%. This suggests that the addition of citric acid can activate the soil, enhancing the solubility and mobility of soil Ni and reducing the adsorption of Ni to soil particles, which is consistent with the results reported previously by Huang et al. (2014) [31]. Furthermore, less variation was observed in the proportions of carbonate, organic, and residual Ni forms, while the content of Ni combined with iron-manganese oxide was reduced from 31.70% to 14.92% with the addition of 200 mg/kg Ni to the soil. In addition, citric acid decreased the soil pH, which may enhance the solubility of soil heavy metals and improve their (bio)availability, thereby reducing the content of iron and manganese oxide-bound Ni [32].

#### 3.3.2. Effect of Malic Acid on Ni Forms

**Figure 7:**
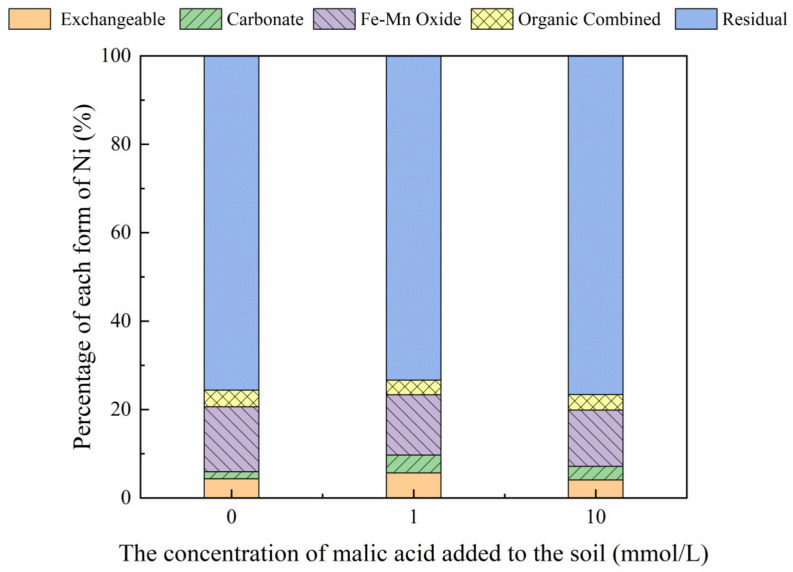
Distribution of different Ni forms after the addition of malic acid (0, 1, 10 mmol/L) to the air-dried mine soil.

The addition of different malic acid concentrations to the reclaimed soil slightly increased the proportion of exchangeable Ni in the soil (Figure 7), whereas the content of Ni combined with Fe-Mn oxide decreased from 14.69% to 12.72%.

**Figure 8:**
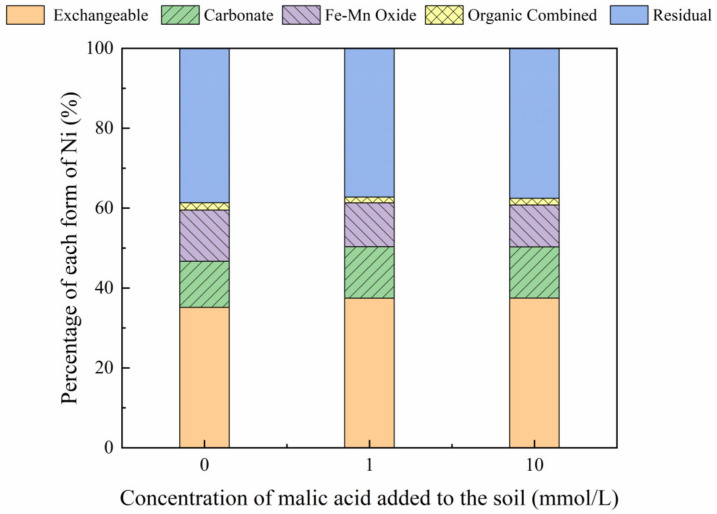
Distribution of different Ni forms in Ni-100 soil after the addition of malic acid (0, 1, 10 mmol/L).

**Figure 9:**
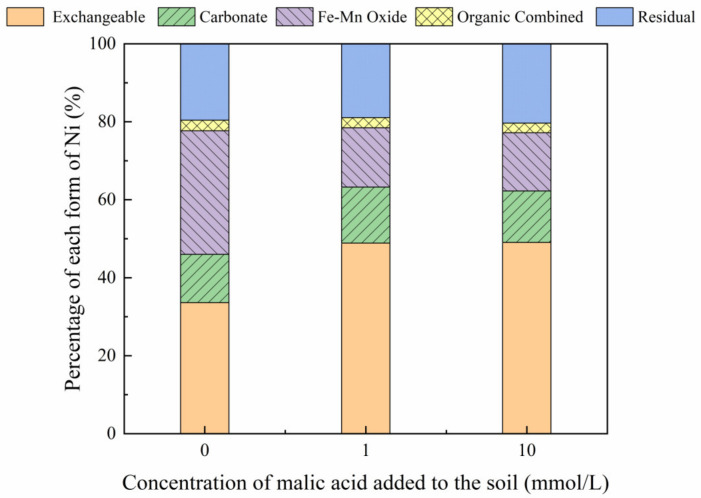
Distribution of different Ni forms in Ni-200 soil after the addition of malic acid (0, 1, 10 mmol/L).

**Figure 10:**
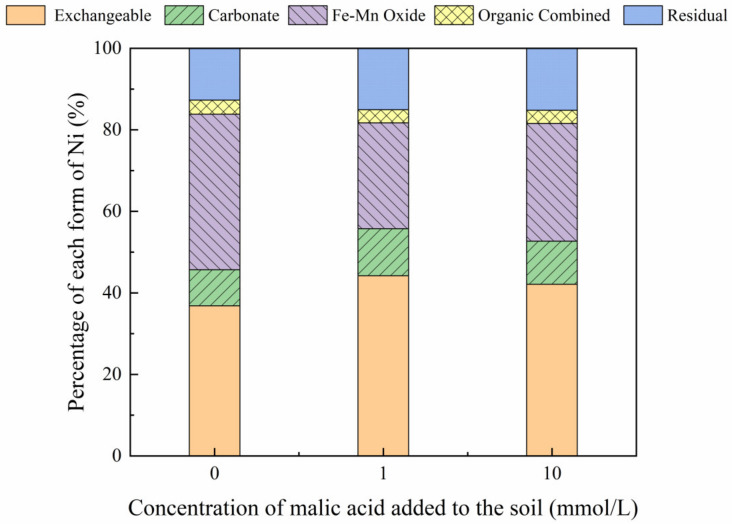
Distribution of different Ni forms in Ni-300 soil after the addition of malic acid (0, 1, 10 mmol/L).

**Figure 11:**
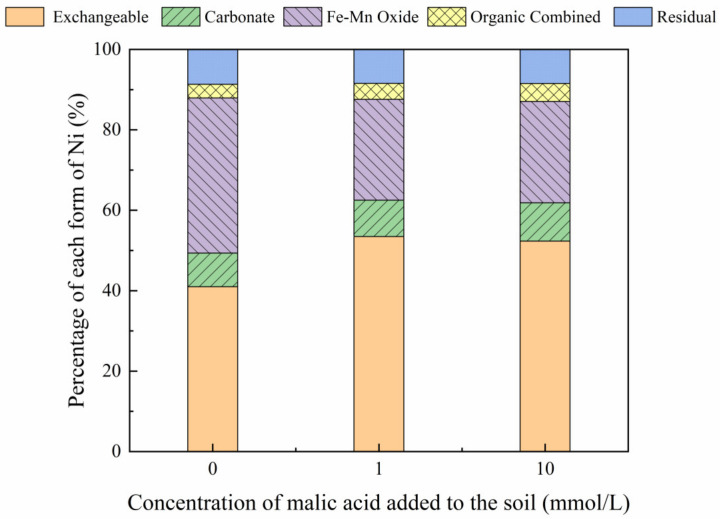
Distribution of different Ni forms in Ni-600 soil after the addition of malic acid (0, 1, 10 mmol/L).

Figure 8, Figure 9, Figure 10 and Figure 11 show that the addition of malic acid caused the exchangeable Ni content to increase significantly from 33.60% to 49.03%, particularly at 200 mg/kg Ni. Due to the increase in malic acid concentration, the complex reaction between heavy metals and organic acids was enhanced, increasing Ni concentration and the electric conductivity of the soil, while also decreasing the soil pH. In addition, malic acid reduced the rate of heavy metal adsorption by the soil, which may result in greater heavy metal availability within the soil [33]. However, the effect of malic acid was less than that of citric acid as citric acid is a tricarboxylic acid, whereas malic acid is a dibasic acid. The addition of organic acids to the soil results in different Ni dissolution rates, with citric acid causing a higher Ni dissolution rate than malic acid. This finding is consistent with the results reported previously [34]. Citric acid not only weakens the adsorption rates of heavy metal ions but also weakens the rate of electrical adsorption. Zheng et al. (2009) [35] showed that the addition of chelating agents (e.g., EDTA, EDDS, and organic acids) to the soil reduces the fraction of Ni bound to Fe and Mn oxides, increases the activity of Ni, and promotes its conversion to the exchangeable Ni form. In addition, the addition of malic acid results in a slight increase in the organic Ni form. The percentage of Ni in the residual state did not change significantly with the addition of malic acid.

### 3.4. Influence of Organic Acids on Dynamic Changes in Soil Ni Forms

**Figure 12:**
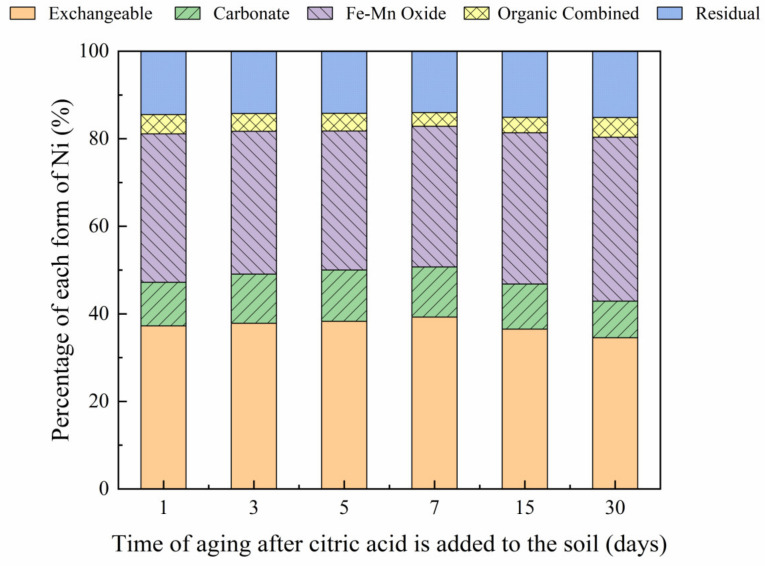
Distribution of different Ni forms at different aging times (1, 3, 5, 7, 15, and 30 days) after the addition of citric acid to Ni-300 mg/kg contaminated soil.

**Figure 13:**
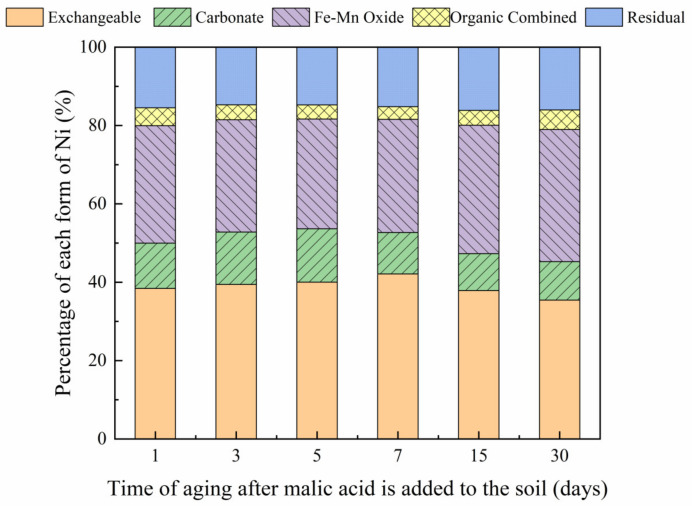
Distribution of different Ni forms at different aging times (1, 3, 5, 7, 15, and 30 days) after the addition of malic acid to Ni-300 mg/kg contaminated soil.

The changes in soil Ni fraction under the influence of the two organic acids, over a 30-day incubation period, are shown in Figure 12 and Figure 13. The addition of citric and malic acids resulted in an initially increasing trend in the proportion of the exchangeable Ni fraction, which subsequently decreased until an equilibrium was reached. Results showed that the addition of citric acid increased the proportion of the exchangeable Ni form from 37.24% to 39.25% from day 1 to day 7, which then gradually decreased over the remaining incubation time, reaching a final proportion of 34.52% on day 30. The same findings were observed following the addition of malic acid with an increase in the proportion of exchangeable Ni forms from 36.52% to 42.11% from day 1 to day 7, which then gradually decreased with time, reaching a final proportion of 35.44% on day 30. These findings may be due to the significant decrease in soil pH following the addition of organic acids that increased the heavy metal activity in the soil [36,37]. However, results revealed a decrease in the concentration of organic acids in the soil, increasing the soil pH, while also decreasing the activity of Ni and the proportion of exchangeable Ni forms in the soil. This finding may be due to the relatively low stability of molecular organic acids [38]. The proportion of Ni in the carbonate-bound state also increased and then decreased with time. However, an increase in malic acid in the soil led to a greater increase in the content of the carbonate combined form than an increase in citric acid. In contrast to the other Ni forms, the proportion of iron-manganese oxide-bound Ni decreased and then increased with further incubation time, reaching a minimum proportion of 31.77% and 28.03% on day 5, in soils containing citric acid and malic acid, respectively, subsequently increasing to 37.47% and 33.73%, respectively, on day 30. The organic form of Ni did not show any significant changes over the first 5 days of incubation. The addition of citric acid resulted in minimum and maximum proportions of combined organic Ni forms of 3.14% and 4.49% on days 7 and 30, respectively; whereas the addition of malic acid resulted in minimum and maximum proportions of 3.22% and 4.99% on days 7 and 30, respectively. Furthermore, results revealed no significant changes in the residual Ni form over the incubation period. Following the addition of malic and organic acids, temporal changes occurred, with Ni transforming from weakly to strongly bound forms in soil, which is consistent with the findings reported previously by Rui et al. (2016) [39].

## 4. Discussion

The reclaimed soil in the study area was filled with coal gangue at the bottom of the coal mining pit, covered with an artificial top layer and then leveled by mechanical rolling [40]. Numerous studies have previously revealed that soil Ni mainly exists in a residual form, in which heavy metals are bound to crystalline silicate minerals such as clay, making it difficult for them to enter the environment under normal conditions [41]. Therefore, residual Ni is chemically stable and minimally affected by environmental factors [42,43], with this form being characterized by low availability, thus presenting a low risk to the surrounding environment and ecosystem. However, the results of this study show that exchangeable Ni and Fe-Mn oxide-bound forms were the major Ni forms in soil following the addition of different Ni concentrations (0–600 mg/kg Ni) [44,45]. The residual fraction of Ni in the soil is converted to an exchangeable state that is more conducive to plant absorption. The associated decrease in proportion of residual Ni suggested that Ni can migrate within the soil as the level of soil Ni contamination increases, with this enhanced mobility subsequently increasing the risk posed by Ni in the soil [46,47]. The addition of organic acids to the soil decreased the soil pH and weakened the adsorption of heavy metals by soil particles [47], thus increasing the (bio)availability of heavy metals. Results showed a negative correlation between soil pH and the proportion of Ni in an exchangeable state, thus favoring plant uptake and increasing (bio)availability [48]. pH reduction led to an increase in the positive charge on the soil's solid phase surface, with hydrogen ions competing with heavy metals for binding sites at pH < 6, leading to heavy metal leaching and inhibiting the adsorption of heavy metals by the soil [49]. The effects of organic acids (malic and citric acids) on Ni activation and soil pH were assessed by monitoring the organic acid leaching in the soil. The results showed that the soil pH was significantly decreased with increasing organic acid concentrations. The addition of organic acids led to an increase in the exchangeable Ni form and a decrease in the Fe-Mn oxide form in the soil, while other Ni forms exhibited insignificant changes [37]. Similarly, Huang et al. (2014) [45] found that with an increase in organic acid, the proportion of exchangeable Ni increased and the Fe-Mn-bound state decreased, supporting the phytoremediation process. Therefore, the addition of different concentrations of Ni and organic acids to the reclaimed soil can change the content, pattern, and (bio)availability of Ni forms in the soil. In fact, organic acids can enhance the activation and migration of soil Ni. This study provides a theoretical basis for the phytoremediation of Ni-contaminated soils, with the exchangeable state content becoming more favorable for phytoremediation and thus less harmful to the soil [50,51]. As low molecular organic acids are biodegradable and exhibit relatively low persistence characteristics in the soil, they are considered environmentally friendly, making them well-suited for use in the reduction in soil contamination. Future experiments will be performed to assess the effects of washing soil with organic acid solutions during remediation.

## 5. Conclusions

The soil Ni forms were significantly affected by the addition of Ni. The results showed that with the increase in exogenous Ni concentration, the morphology of Ni in soil was transformed, in the following order, exchangeable state > iron-manganese oxide combined > residual > carbonate combined > organic combined. Since the exchangeable Ni form was the dominant form in the soil, the activity of Ni in the soil increased significantly.

The addition of citric acid and malic acid to soil increased the (bio)availability of Ni. In addition, the degree of Ni activation increased with increasing organic acid concentrations. The increases in exchangeable Ni and Ni carbonate combined forms in the soil were lower when using malic acid than those obtained using citric acid under the same conditions. Moreover, citric acid exerted a stronger activation effect than malic acid.

The results of the incubation experiment revealed a gradual transformation of soil Ni from loosely bound Ni into strongly bound Ni in the presence of citric and malic acids.

Due to the addition of organic acids, the proportion of exchangeable forms of Ni in the soil increased, increasing the (bio)availability of the soil and making Ni more easily absorbed by plants, thus reducing the toxicity of heavy metals in the soil. In addition, compared to malic acid, citric acid has better activation properties and may be useful for the remediation of nickel-contaminated soils, improving the effectiveness of phytoremediation techniques.

## Figures and Tables

**Table 1:** Concentration and type of organic acid added to Ni-contaminated reclaimed soil samples.

Types of Organic Acids	Molecular Formula	Molecular Weight	Add Concentration (mmol/L)		
Citric acid	C_6_H_8_O_7_	192.14	0	1	10
Malic acid	C_4_H_6_O_5_	134.09	0	1	10

**Table 2:** Measured concentrations of total nickel (all fractions) in spiked remediation soil samples and the range of relative deviation.

Measurement Object	Spiked Concentration of Ni-Contaminated Soil (mg/kg)	Measured Mean Total Ni Concentration (mg/kg)	Relative Deviation (%)
A	100	95.31	1.36
B	200	236.56	2.69
C	300	301.66	0.55
D	600	563.69	3.05

**Table 3:** Physicochemical properties of the reclaimed soil sample.

Capacity (g/cm^3^)	pH	Organic Matter (g/kg)	Available Potassium (mg/kg)	Available Phosphorus (mg/kg)	Available Nitrogen (mg/kg)
1.33	7.8	4.13	191.03	10.41	28.98

## Data Availability

The datasets used and/or analyzed during the current study are available from the corresponding author on reasonable request.
